# Single-port robotic colorectal surgery: a scoping review of outcome reporting and future directions for standardisation

**DOI:** 10.1007/s11701-026-03402-9

**Published:** 2026-04-21

**Authors:** Pia Borgas, Taner Shakir, Nader Francis, Deborah S. Keller

**Affiliations:** 1 Faculty of Medicine, Clarunis, Universitäres Bauchzentrum, University of Basel, Basel, Switzerland; 2 Department of Surgery and Interventional Science, University College London, London, UK; 3 The Griffin Institute, London, UK; 4 Department of Surgery, Mayo Clinic, Phoenix, AZ USA; 5 School of Biological and Health Systems Engineering, Arizona State University, Tempe, AZ USA

**Keywords:** Robotic surgery, Robotic assisted surgery (RAS), Colorectal surgery (CRS), Single port robotic surgery (SP, SPr)

## Abstract

**Background:**

Single-port (SP) robotic-assisted surgery is the latest development in minimally invasive colorectal surgery and may offer advantages such as improved cosmesis, reduced pain, and shorter hospital stay. However, evidence remains fragmented, and inconsistent outcome reporting limits comparison across studies and meaningful meta-analysis. This scoping review evaluated outcome reporting in SP robotic colorectal surgery to identify gaps and inform standardisation.

**Methods:**

A scoping review was conducted in accordance with PRISMA-ScR guidelines. Ovid MEDLINE, Embase, and the Cochrane Library were searched from inception to January 2026. Studies reporting clinical outcomes of SP transabdominal robotic colorectal surgery or comparing SP with multi-port (MP) approaches were included. Data extraction assessed reporting completeness across patient demographics, operative details, intraoperative outcomes, postoperative recovery, and long-term follow-up.

**Results:**

Nineteen studies met inclusion criteria, comprising 992 patients, of whom 610 underwent SP robotic resection. Eight studies were comparative and eleven non-comparative. Most were single-centre retrospective series from high-volume centres in the United States and South Korea. Outcome reporting was highly heterogeneous. Operating time, complications, and conversion rates were most consistently reported. In contrast, postoperative and patient-centred outcomes were inconsistently captured, including pain scores (4/19 studies), return to theatre (2/19), and follow-up interval (5/19). All studies reported complications, but only 9/19 used standardised grading systems. No study assessed health economic outcomes or cost-effectiveness.

**Conclusion:**

Outcome reporting in SP robotic colorectal surgery remains inconsistent and focused mainly on technical feasibility. Standardised core outcome sets are needed to support robust comparison, pooled analysis, and evidence-based adoption.

## Introduction

Minimally invasive colorectal surgery (CRS) emerged in response to the substantial morbidity associated with traditional open procedures [1, 2]. Open approaches necessitated large incisions, leading to significant postoperative pain, prolonged ileus, higher wound complication rates, and delayed return to normal activity [3–6]. The advent of laparoscopic colectomy in the early 1990s demonstrated that smaller incisions could reduce surgical trauma, translating to faster recovery, decreased postoperative pain, shorter length of stay, and improved quality of life, while maintaining equivalent oncologic outcomes in cancer cases [5–7]. Despite these advantages, conventional laparoscopy presented notable limitations, including reduced degrees of freedom, two-dimensional visualization, and ergonomic strain on the surgeon, which restricted its widespread adoption for technically demanding operations [8]. Robotic-assisted surgery (RAS) was introduced to overcome these barriers, offering high-definition three-dimensional visualization, articulating instruments that mimic wristed motion, and improved ergonomics [9]. As such, the rise in overall MIS over the last decade has been driven largely by expansion of RAS. The RAS platforms continue to evolve with the goal of improving both patient outcomes and surgeon performance.

RAS has traditionally relied on a multi-port (MP) approach, requiring multiple small incisions to accommodate the robotic arms and instruments. In line with the broader surgical trend toward reducing invasiveness and improving cosmetic outcomes, single-port (SP) RAS was developed as a next step in the evolution of minimally invasive techniques. SP robots were designed especially for either natural orifice surgery or to address the limitations of single port laparoscopic surgery. SP surgery utilizes a single, larger incision to introduce a multi-channel port through which all robotic instruments are deployed [10]. While innovative, commercially available SP platforms have inherent technical constraints compared to MP systems, including fewer degrees of freedom, reduced intra-abdominal triangulation, and the absence of integrated energy and stapling devices, compared to MP systems [11]. Nevertheless, SP RAS has demonstrated unique advantages in select specialties, such as urology [12]. In CRS, SP RAS remains relatively novel and continues to evolve [10]. Despite the growing interest in SP robotic CRS, studies assessing safety and efficacy have heterogeneous reporting outcomes preventing meaningful meta-analysis [13]. Given these limitations, a comprehensive synthesis of available evidence is warranted to clarify its role and potential in the field.

The aim of this scoping review was to systematically evaluate the current literature on SP robotic colorectal surgery, assess the completeness and consistency of outcome reporting, identify methodological and reporting gaps, and summarise the clinical outcomes reported in the current literature so as to provide context to the aforementioned gaps. Furthermore, the aim was to define outcome domains to guide future research and establish standardised reporting criteria.

## Methods

This scoping review was conducted in accordance with the Preferred Reporting Items for Systematic Reviews and Meta-Analyses extension for Scoping Reviews (PRISMA-ScR) guidelines [14]. A predefined protocol outlined inclusion, the review question, eligibility criteria, and methodological approach.

### Search strategy

A comprehensive search of Ovid MEDLINE, Embase and the Cochrane Library was performed, from database inception to January 2026. The strategy was developed in consultation with a medical librarian and incorporated both controlled vocabulary (MeSH/Emtree) and free-text keywords related to “colorectal surgery,” “robotic surgery,” “single-port,” and “multi-port”. Boolean operators, adjacency searching, and truncation were applied to maximise sensitivity and specificity. Full search strategies are provided in Appendix 1. After removal of duplicates, 88 unique records were identified for screening.

### Eligibility criteria

Eligible studies included primary research articles (randomised controlled trials, prospective and retrospective cohort studies, case series, and case reports) reporting clinical outcomes of:

- SP transabdominal robotic colorectal surgery; or.
- Comparative studies of SP versus multi-port (MP) robotic colorectal approaches.

Studies were included irrespective of patient age or pathology (benign or malignant).

Exclusion criteria were non-English publications, review articles, conference abstracts without full data, preclinical/cadaveric studies, natural orifice surgery such as transanal total mesorectal excision (TaTME) surgery without an abdominal component, and reports that did not provide procedure-specific outcomes for colorectal surgery.

### Robotic platforms considered

Commercially available robotic surgical systems designed for soft-tissue procedures were included. For MP systems, this included the da Vinci Si, Xi, and X platforms (Intuitive Surgical, Sunnyvale, CA, USA), Medtronic Hugo RAS System (Medtronic, Minneapolis, MN, USA), CMR Surgical Versius Surgical System (CMR Surgical, Cambridge UK) and SS Innovations SSi Mantra Surgical Robotic System (SS Innovations, Haryana, India). These systems employ multiple trocar sites for independent robotic arms and camera systems. For SP systems, this included the da Vinci SP platform (Intuitive Surgical) and Surgerii Robotics SHURUI Surgical System (Beijing Surgerii Robotics Company Limited, Beijing, China). SP platforms utilise a single multichannel port accommodating a flexible camera and articulating instruments through one incision.

### Study selection

**Fig. 1:**
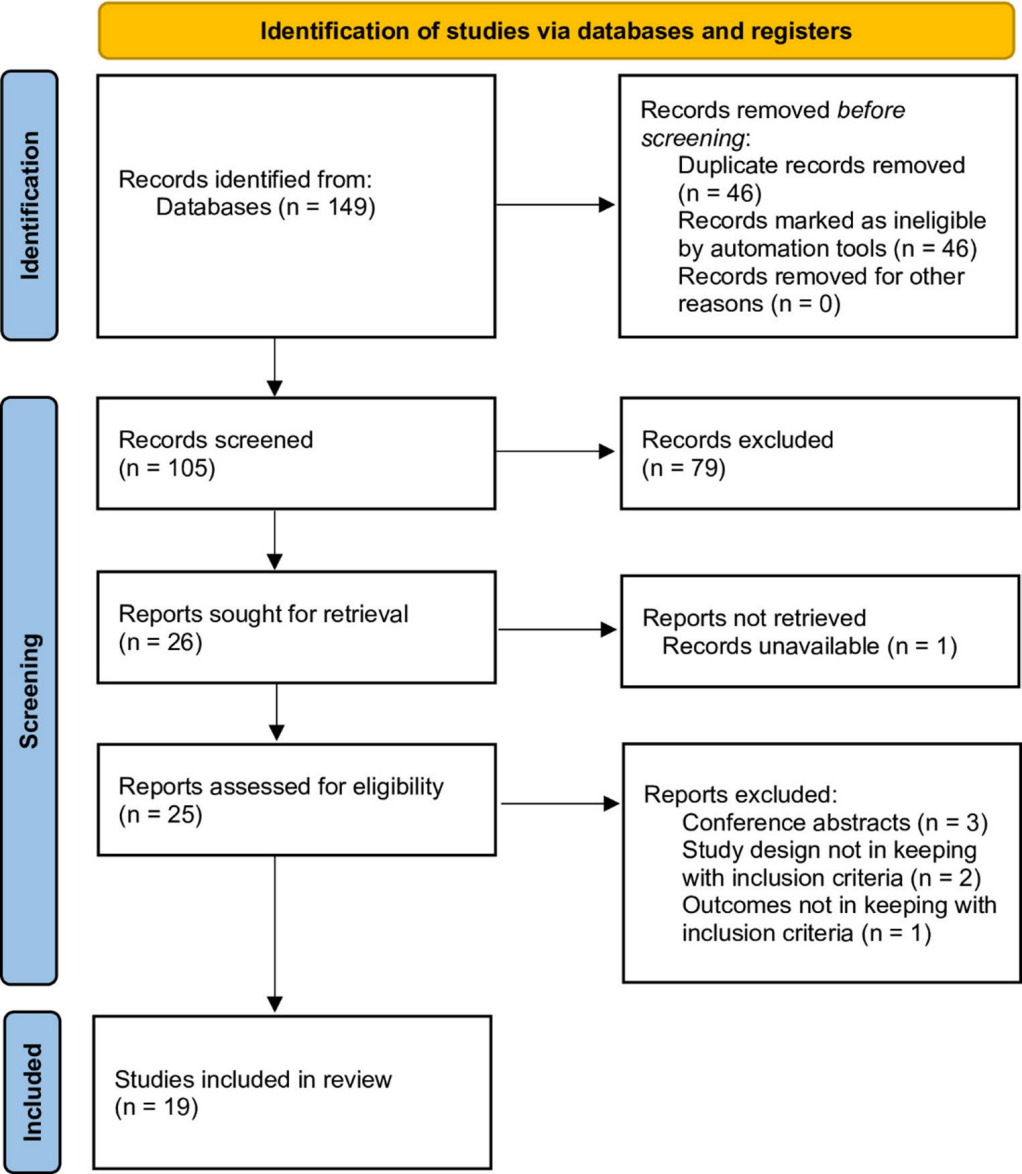
PRISMA-ScR Flow Diagram for Included Studies. Source: Page MJ, et al. BMJ 2021;372:n71. doi: 10.1136/bmj.n71.

Two reviewers (PB, TS) independently screened titles and abstracts, followed by full-text review of potentially eligible studies. Discrepancies were resolved through consensus or third-reviewer adjudication (DK). Reasons for exclusion were documented. The selection process is illustrated in a PRISMA-ScR flow diagram (Fig. 1).

### Data extraction

To assess the level of consistency across studies, data extraction captured whether key variables were reported, allowing descriptive assessment of reporting completeness rather than a formal quantitative measure of heterogeneity. Template was used to capture all included studies, recording study characteristics (year, country, study design, sample size, robotic platform used), patient demographics (age, sex, body mass index [BMI], comorbidities, benign/malignant pathology, neoadjuvant/adjuvant therapy rate), operative details (procedure, port configuration and type, docking time, conversion rates to laparoscopic and open approaches), intraoperative details (operating time, incision length, lymph node yield, margin status, blood loss, intra-operative complications) and postoperative details (length of stay, postoperative morbidity and mortality (including Clavien–Dindo classification), bowel recovery, readmission, pain scores, return to theatre, and recurrence (including local and systemic), and total follow up duration). These outcome domains were selected a priori based on established reporting frameworks for minimally invasive colorectal surgery, including the CONSENSUS-CRC core outcomes for colorectal surgery [15] and the RoboCOS core outcome set for robotic-assisted surgery [16], supplemented by variables consistently reported in prior comparative studies of SP and MP robotic approaches. The domains and outcomes were considered to represent a minimum dataset necessary to enable meaningful assessment of reporting completeness and to identify gaps relevant to standardisation.

### Assessment of reporting completeness

To evaluate consistency in reporting across studies, we recorded whether key variables were reported at all, regardless of the specific values. This descriptive assessment allowed identification of gaps in outcome reporting and highlighted areas where standardisation is lacking. Formal risk of bias or quality assessment was not performed, consistent with scoping review methodology, but this descriptive mapping serves to assess reporting completeness rather than study quality.

### Risk of bias and data synthesis

Consistent with scoping review methodology, formal risk of bias assessment was not performed. Given substantial heterogeneity in study design, patient selection, and outcome definitions, meta-analysis was not feasible.

Findings were synthesised narratively, with descriptive statistics used where appropriate. Reporting frequency was analysed to identify patterns of consistency and omission.

## Results

### Search findings

A total of 19 studies met the inclusion criteria and were included in this scoping review. Of these, eight were comparative studies, including three comparing SP robotic surgery to laparoscopic surgery [17–19] and five comparing SP to MP robotic surgery [20–24]. The remaining eleven studies were non-comparative, focusing SP-only series without a comparison group [25–35].

### Study characteristics

18 included studies were single-centre and 1 was multi-centre [19]. Designs included six case series [25, 26, 28, 29, 31, 35], eleven retrospective cohort studies [17–24, 27, 33, 34], and two phase II clinical trial reports from the same institution [30, 32]. Sample sizes ranged from 2 to 181 patients. Across all studies, 992 patients were included, of whom 610 underwent SP robotic resection. Sixteen studies utilised the da Vinci SP platform. Two studies used the SHURUI system [23, 33], and one used the da Vinci Si [22]. There was marked geographic clustering, with most publications originating from two high-volume centres in the United States and South Korea. Overall, the included studies were conducted across ten different centres. Table 1 summarises the study characteristics.

**Table 1:** Study demographics and design

Author & Year	Country	Study design	Single/Multi-Centre	Device Name		Study Period	*N*		
				SP	Control		Total	SP	control
Kim et al. 2024	Korea	Retrospective cohort study	Single	Da Vinvi SP	Da Vinci Xi	Jul 2020 - Jun 2022	84	42	42
Jeong et al. 2023	Korea	Retrospective cohort study	Single	Da Vinci SP	Da Vinci Xi	Jan 2020 - Jan 2021	26	13	13
Song et al. 2021	Korea	Case series	Single	Da Vinci SP	-	July 2020- Sept 2020	5		
Kim et al. 2021	Korea	Case series	Single	Da Vinci SP	-	July 2020 - sept 2020	5		
Picciariello et al. 2023	Korea	Case series	Single	Da Vinci SP	-	Dec 2021 – Jul 2022	2		
Kim et al. 2023	Korea	Retrospective cohort study	Single	Da Vinci SP	Laparoscopy	Feb 2019 - August 2022	140	43	97
Kim et al. 2023	Korea	Retrospective cohort study	Single	Da Vinci SP	-	March 2019-Sept 2021	50		
Bae et al. 2022	Korea	Retrospective cohort study	Single	Da Vinci SP	Laparoscopy	Aug 2014 - Jan 2016	97	36	61
Cho & Kim 2024	Korea	Case series	Single	Da Vinci SP	-	May 2023 - Dec 2023	10		
Piozzi et al. 2023	Korea	Case series	Single	Da Vinci SP	-	Nov 2020 - Dec 2021	13		
Noh et al. 2025	Korea	Retrospective cohort study	Multi	Da Vinci SP	Laparoscopy	March 2019 – April 2023	181	95	86
Marks et al. 2023	USA	Phase II clinical trial	Single	Da Vinci SP	-	Oct 2018 - Aug 2021	93		
Marks et al. 2020	USA	Case series	Single	Da Vinci SP	-	Oct-18	2		
Marks et al. 2022	USA	Phase II clinical trial - full text not available	Single	Da Vinci SP	-	Oct 2018 - Aug 2021	133		
Sarin et al. 2024	USA	Retrospective cohort study	Single	Da Vinci SP	Da Vinci Xi	May 2022 – Nov 2022	30	5	25
Chang et al. 2020	Taiwan	Retrospective cohort study	Single	Da Vinci Si	Da Vinci Si	Jan 2017 - Jul 2019	40	20	20
Guo et al. 2023	Shanghai	Retrospective cohort pilot study	Single	SR-ENS-600	-	March 2022 - Sept 2022	7	7	7
Huang et al. 2025	Shanghai	Retrospective cohort study	Single	Da Vinci SP	-	Oct 2023 – July 2024	15		
Shi et al. 2025	China	Retrospective cohort study	Single	Jingfeng SP1000 and SR-ENS-600	Da Vinci Xi		59	21	38

SP: Single Port; Xi/Si: Da Vinci multi-port systems

### Patient demographics

Table 2 summarises the patient demographics across the included studies. There was variability in the male-to-female ratio, with one study reporting gender-matched cohorts [31].

**Table 2:** Patient population characteristics

Author & Year	Sex		Mean age		Mean BMI		Benign/malignant	Rate of pre op chemo/RT (%)	
	Male (%)	Female (%)	Single port	Control	SP	Control		SP	Control
Kim et al. 2024	66	33	62.5	61.5	24.4	24.7	Malignant	61.9	61.9
Jeong et al. 2023	54	46	53.2	59.0	23.4	24.3	Malignant	38.5	38.5
Song et al. 2021	80	20	69.0	-	24.6	-	Malignant	40.0	
Kim et al. 2021	60	40	57.0	-	23.8	-	Malignant	40.0	
Picciariello et al. 2023	0	100	72.0	-	21.0	-	Malignant	100	
Kim et al. 2023	28	72	58.8	70.6	23.4	24.3	Malignant		
Kim et al. 2023	52	48	59.0	-	24.0	-	Malignant (22%) & benign (78%)	23.0	
Bae et al. 2022	47	62	62.0	67.0	24.6	24.0	Malignant & benign	52.8	49.2
Cho & Kim 2024	40	60	63.5	-	22.9	-	Malignant	40.0	
Piozzi et al. 2023	69	31	56.0		22.8	-	Malignant		
Noh et al. 2023	43	52	59.3	63.4	63.4	23.8	Malignant		
Marks et al. 2023	42	58	59.7	-	27.5	-	Malignant & benign	41.0	
Marks et al. 2020	50	50	56.0	-	26.0	-	Benign		
Marks et al. 2022	42	58		-			Malignant & benign		
Sarin et al. 2024	-	-	-	-	-	-	Malignant		
Chang et al. 2020	45	55	62.4	63.8	24.9	23.6	Malignant		
Guo et al. 2023	7	14	60.6	-	24.6	-	Malignant		
Huang et al. 2025	48	52	62.7	64.2	23.8	24.3	Malignant		
Shi et al. 2025	53.3	46.7	-	-	-	-	Benign (20%) and malignant (80%)		

SP: Single Port; BMI: Body Mass Index; Chemo/RT: Chemotherapy/Radiotherapy

BMI was relatively homogeneous (mean ≈ 24 kg/m²) across the SP and control groups.

Most studies (*n* = 13) evaluated malignant disease exclusively [17, 19–25, 28, 29, 33, 35]. One focused on benign pathology [31]; five included mixed indications [18, 27, 30, 32, 34]. Cancer staging was inconsistently reported, with variable TNM stratification and limited stage-specific outcome analysis. Similarly, the use of neoadjuvant or adjuvant therapies was inconsistently documented, but on average received by 49% of SP groups and 50% of the control groups.

### Operative technique

Procedure types varied across studies (right hemicolectomy, left hemicolectomy, anterior resection, low anterior resection). Port platforms included Uniport (*n* = 9), Glove port (*n* = 5), and the GelPoint access system (*n* = 4). Port configuration and location were generally tailored to tumour location and procedure type, typically involving a single larger incision for the robotic port and one additional laparoscopic assistant port. It is worth noting at the time of this review, there was no robotic stapler available for the SP platform. Five studies reported the use of extra ports beyond this standard setup [21, 26, 28, 30, 32]. The specimen extraction site was detailed in four studies; in all four, the SP incision was used for specimen extraction to minimise additional wounds [20–22, 26]. Depending on tumour location, extraction sites included a right lower quadrant transverse incision, Pfannenstiel incision, or a mini laparotomy. Docking time (reported in 10 studies) ranged from < 3 min to 14 min (mean ≈ 9 min). Conversion rates were reported in 17 studies, ranging from 0 to 3%, except one reporting an outlier 29% conversion to laparoscopy [33]. Table 3 outlines the operative techniques reported across studies.

**Table 3:** Operative Technical Specifications. Comparison of procedural types, docking efficiency, and port configurations

Author & Year	Procedure		Mean Docking Time (Mins/Secs)		Port Type Uniport	Port set-up			Conversion to Lap Rate (%)		Conversion to Open Rate (%)	
	SP	Control	SP	MP		SP	Need for further ports (%)	Control	SP	MP	SP	MP
Kim et al. 2024	Low anterior resection + TME 71.4% Intersphincteric resection 28.6%	Low anterior resection 7 + TME 1.4% Intersphincteric resection 28.6%	-	-	Uniport	TME: - 1 × 4 cm incision in RLQ for Uniport which also served as specimen extraction site - 1 × 5 mm assistant port in RUQ	0.0	4 × 8 mm ports in oblique linear line from right ASIS to LUQ 1 x assistant port in LUQ Extraction through 4–6 cm extension of RLQ trocar	0.0	0.0	0.0	0.0
Jeong et al. 2023	Low anterior resection + TME 61.5% Intersphincteric resection 38.5%	Low anterior resection + TME 76.9% Intersphincteric resection 23.1%	-	-	Uniport	TME: - 1 × 4 cm transverse incision in RLQ for Uniport which also serves as specimen extraction site - 1 × 12 mm assistant port and 2 × 5 mm assistant ports	15.0	1 × 12 mm supraumbilical port for camera 3 × 8 mm robotic working ports 1–2 x additional laparoscopic assistant ports	-	-	1.7	-
Song et al. 2021	Right hemicolectomy 100%	-	4 mins 40 secs	-	Uniport	4 cm Pfannenstiel incision at 4 finger-widths above the symphisis pubis through which the Uniport device was placed Additional 5 mm laparoscopic assistant port in LLQ	-	-	0.0	-	0.0	-
Kim et al. 2021	Low anterior resection + TME 100%	-	4 mins 20 sec	-	Uniport	1 × 4 cm incision in RLQ for Uniport 2 × 5 mm assistant port in RUQ 4 cm mini laparotomy for specimen extraction	25.0	-	0.0	-	0.0	-
Picciariello et al. 2023	APR	-	4 min 10 s	-	Uniport	3 cm transverse incision in left lower quadrant at colostomy site, for Uniport insertion 5 mm laparoscopic assistant port in epigastrium	-	-	-	-	-	-
Kim et al. 2023	Right hemicolectomy (12%) Transverse colectomy (0%) Left hemicolectomy (6%) Anterior resection (22%) Low anterior resection (3%)	Right hemicolectomy (42%) Transverse colectomy (1%) Left hemicolectomy (8%) Anterior resection (33%) Low anterior resection (13%)	-	-	Glove port (44%) and Uniport (56%)	Variable depending on operation type - not described in detail	-	1 × 10 mm infraumbilical (extended for specimen extraction) + 3 x ports according to tumour location	0.0	N/A	0.0	2.1
Kim et al. 2023	Right colectomy (12%) Sigmoid colectomy (2%) Right colectomy + CME + CVL (18%) Left colectomy + CME + CVL (8%) Sigmoid colectomy + CME + CVL (28%) Low anterior resection + TME (32%) 28% sigmoid colectomy + CME + CVL, 12% right colectomy, 2% sigmoid colectomy	-	14 min	-	Glove port (44%) and Uniport (56%)	Assistant lap port for LAR in RLQ if ileostomy was planned and suprapubic if no ileostomy planned	-	-	0.0	-	0.0	-
Bae et al. 2022	-	-	-	-	Glove port	25 mm vertical incision through umbilicus for single port insertion (single-Site port or Glove port) Additional 12 mm incision for conventional robotic port in RLQ	-	2 × 12 mm at umbilicus (camera) and RUQ (working port) 3 × 5 mm in each remaining quadrant periumbilical or transumbilical extension for extraction site	0.0	N/A	3.1	0.0
Cho & Kim 2024	Right hemicolectomy (20%) Anterior resection (50%) Low anterior resection (10%) Ultra-low anterior resection (20%)	-	< 3 min	-	Glove port	Right hemi - transumbilical incision for glove port left-sided colectomy - transumbilical incision for glove port ULAR - circular incision at RLQ at ileostomy site for glove port	1.0	-	-	-	-	-
Piozzi et al. 2023	Intersphincteric resection (54%) Right colectomy (38%) Transverse colectomy (8%)	-	ISR: 7 mins RC/TC: 5 min	-	Uniport	ISR: 30 mm transverse RLQ for Uniport system + 12 mm RUQ port RC/TC: 30 mm Pfannenstiel incision for Uniport system + 12 mm laparoscopic port in LLQ	-	-	0.0	0.0	0.0	0.0
Noh et al. 2025	Colorectal resections		-	-	-	Right hemi: transumbilical or Pfannenstiel incision to insert single port. Anterior resection: tranumbilical or right lower quadrant incision	-	-	0.0	4.7	2.1	2.3
Marks et al. 2023	Left colectomy (23.65%) Low anterior Resection (12.9%) Completion proctectomy (12.9%) Right hemi (8.6%) Rectopexy (2.15%) Hartmann’s (1.07%) TATA (32.25%)APR (6.45%)	-	14.87 min (colectomies) 20.6 min (rectal cancer cases)	-	GelPoint access platform	-	5.9	-	0.0	-	2.2	-
Marks et al. 2020	Left colectomy (100%)	-	13 min	-	GelPoint access platform	4 cm transverse rectus abdominus muscle-splitting incision in right lower quadrant for singe port	0.0	-	0.0	-	0.0	-
Marks et al. 2022	Right hemi + anterior resection	-	-	-	GelPoint access platform	-	0.0	-	-	-	0.0	-
Marks et al. 2022	Right hemi, anterior resection, right colectomies, taTME, TAMIS, APR, proctocolectomy, rectopexy, Hartmann’s reversal				GelPoint access platform		3.1	-	3.0	-	0.0	-
Sarin et al. 2024	Right hemicolectomy					Pfannenstiel incision	-	-	0.0	0.0	0.0	0.0
Chang et al. 2020	Right hemicolectomy (30%) Left hemicolectomy (5%) Sigmoidectomy (0.65%)	Right hemicolectomy (20%) Left hemicolectomy (10%) Sigmoidectomy (70%)	13 min	13 min	Glove port	4–7 cm umbilical incision for Gloveport and specimen extraction 1x additional assistant port	-	-	0.0	0.0	0.0	0.0
Guo et al. 2023	Sigmoid colectomy (29%) Low anterior resection (43%) Right hemi (29%)	-	12 min	-	-	4 cm single incision through right rectus Abdominis at umbilical level for single port additional 12 mm RIF port	-	-	29.0	-	0.0	-
Huang et al. 2025	Proctectomy, Sigmoidectomy and total colectomy	-	-	-	-	-	-	-	0.0	-	0.0	-
Shi et al. 2025	Colorectal resections		-	-	-	-	-	-	0.0	0.0	0.0	0.0

SP: Single Port; MP: Multi-port; TME: Total Mesorectal Excision; LAR: Low Anterior Resection; ISR: Intersphincteric Resection; APR: Abdominoperineal Resection; CME/CVL: Complete Mesocolic Excision/Central Vascular Ligation

### Intra-operative outcomes

Mean operative time ranged from 119 to 357 min, often without procedure-specific stratification. The mean total incision length was generally shorter in the SP robotic approach compared to MP techniques; however, reporting across studies was inconsistent. Lymph node yield (≈ 22 nodes SP; ≈21 nodes MP) and estimated blood loss (≈ 54 ml SP; ≈40 ml MP) were among the most consistently reported outcomes. In contrast, reporting on distal resection margins was more variable; some studies quantified the distance in centimetres, while others simply stated whether margins were positive or negative. Positive margins were rare; they were observed in only one patient within the SP cohort [21] and in two patients across two separate studies in the control cohort [21, 28]. Intra-operative complications were not systematically reported in any study. Table 4 outlines intra-operative details.

**Table 4:** Intra-operative performance and pathology

Author & Year	Mean Operating Time (Minutes)		Mean Lymph Node Harvest		Mean Total Incision Length (cm)		Blood Loss (ml)		Length of Distal Resection Margin (Drm) (cm)		Positive Margins (*N*)	
	SP	Control	SP	Control	SP	Control	SP	Control	SP	Control	SP	Control
Kim et al. 2024	160	200	17.8	19.8	4.0	5.4	27	32	2	2	0	1
Jeong et al. 2023	180	172	26.0	24.5	-	-	20	30	-	-	1	1
Song et al. 2021	160	-	41.0	-	-	-	18	-	-	-	0	-
Kim et al. 2021	195	-	26.0	-	-	-	15	-	Negative	-	0	-
Picciariello et al. 2023	165	-	-	-	-	-	18	-	Negative	-	0	-
Kim et al. 2023	232	204	22.1	23.4	-	-	60	71	Negative	Negative	0	0
Kim et al. 2023	263	-	18.0	-	6.0	-	50	-	47	-	0	-
Bae et al. 2022	232	155	15.0	18.0	5.0	8.0	-	-	4	4	-	-
Cho & Kim 2024	222	-	-	-	3.3	-	148	-	-	-	-	-
Piozzi et al. 2023	280	-	21.0	-	-	-	< 50	-	2	-	0	-
Noh et al. 2025	180	181	10.8	24.9	4.7	4.6	33.7	72.1	9.3	9.2	-	-
Marks et al. 2023	357	-	24.0	-	4.5	-	50	-	Negative	-	0	-
Marks et al. 2020	306	-	-	-	4.3	-	60	-	-	-	-	-
Marks et al. 2022	-	-	30.0	-	4.9	-	82	-	Negative	-	0	-
Marks et al. 2022			18.0		5.5	-		-	Negative	-	0	-
Sarin et al. 2024	-	-	-	-	-	-	-	-	-	-	0	0
Chang et al. 2020	186	193	19.6	22.5	5.5	9.5	10	25	-	-	-	-
Guo et al. 2023	237	-	12.0	-	3.9	-	206	-	5	-	0	-
Huang et al. 2025	119	-	13.5	-	-	-	10	-	3	-	0	-
Shi et al. 2025	299	228	14.8	13.3	-	-	14	10	-	-	-	-

SP: Single Port; DRM: Distal Resection Margin

### Post-operative outcomes

The postoperative follow-up interval was only reported by 5 studies, limiting oncological interpretation [18, 27–29, 32]. Post-operative pain scores (*n* = 4) and return to theatre (*n* = 2) were infrequently reported. Mean time to bowel recovery (first flatus) was reported in 63% of the studies, with a mean of 2 days post-operatively in the SP group. Only two studies provided comparative data for the MP group [19, 27]. The length of stay was reported in 15 studies (mean 7 days SP; 8 days MP). However, interpretation is limited by healthcare system variability. All studies commented on the rate of post-operative complications; however, the level of detail was inconsistent. Recurrence rates were mentioned in six studies, though meaningful interpretation was limited by significant variability in follow-up duration [27, 29, 30, 32, 33]. Complications were universally reported, and nine studies provided Clavien–Dindo grading [18, 23, 25–29, 32, 34]; the remaining reported only an overall complication rate, making it difficult to assess the severity of complications. Table 5 summarises the reported post-operative outcomes.

**Table 5:** Post-operative outcomes and recovery

Author & Year	All Complications (%)		Anastomotic Leak (*N*)		Clavien Dindo Grade (% of Total Complications)	Pain Score	Return to Theatre		Length of Stay (Days)		Time to Flatus / Faeces (Days)		Recurrence (%)	Follow up period (months) -
	SP	Control	SP	Control			SP	Control	SP	Control	SP	Control		
Kim et al. 2024	12	16.6	1	2	-	-	-	-	6	7	-	-	-	-
Jeong et al. 2023	8	23.1	1	3	-	-	-	-	7	8	-	-	-	-
Song et al. 2021	25	-	0	-	I (100)	Reported by post-OP day	-	-	7	-	2	-	-	-
Kim et al. 2021	25	-	-	-	III (100)	-	-	-	7	-	-	-	-	-
Picciariello et al. 2023	0	0	0	0	-	-	-	-	5	-	-	-	-	-
Kim et al. 2023	16	20.6	-	-	-	-	-	-	8	9	2	3	-	-
Kim et al. 2023	10	-	0	-	I-II	-	0	-	7	-	2	-	4	3
Bae et al. 2022	17	23	0	3.3	I-III		-	-	9	9	-	-	-	3
Cho & Kim 2024	10	-	-	-	II (100)	-	-	-	6	-	-	-	-	4.6
Piozzi et al. 2023	31	-	0	-	I-II	-	-	-	7 ISR 5 RC/TC	-	2 ISR 2 RC/TC	-	2 patients had systemic recurrence (?due to advanced stage)	9 ISR 11 RC/TC
Noh et al. 2025	17	14	0	1	-	Reported by Post-OP day	-	-	6	7	3	3	-	-
Marks et al. 2023	27	-	1	-	-	-	-	-	5	-	2	-	0 (20mo f/u)	-
Marks et al. 2020	0	-	0	-	-	0	-	-	3	-	2	-	-	-
Marks et al. 2022	10	-	1	-	IV	-	-	-	-	-	2	-	0 (5.6mo f/u)	5.6
Marks et al. 2022		-	0.78	-							2		0 (15mo f/u)	-
Sarin et al. 2024	20	12	-	-	-	Reported by post-OP day	0	0	-	-	-	-	-	-
Chang et al. 2020	0	0	0	0	-	-	-	-	8	9	-	-	-	-
Guo et al. 2023	0	-	0	-	-	-	-	-	-	-	3	-	0	-
Huang et al. 2025	3	-	2	-	II	Reported by post-OP day	-	-	12	-	2	-	-	-
Shi et al. 2025	62	76	0	0	I-II	-	-	-	-	8	10	-	-	-

SP: Single Port; CD: Clavien-Dindo Grade; ISR: Intersphincteric Resection; RC/TC: Right Colectomy/Transverse Colectomy; f/u: Follow-up

### Reporting frequency of key outcomes

The frequency with which each outcome variable was reported is summarised in Table 6. Reporting was most consistent for operating time, complication rates, or conversion rates, while postoperative pain, return to theatre and long-term follow-up were rarely documented.

**Table 6:** Reporting frequency of key variables

Variable reported	Number of studies	% of total (*n* = 19)
**Operative technique**		
Docking time	10	53
Need for additional ports	10	53
Conversion rate (to laparoscopy or open)	16	84
Port type/configuration	15	79
Specimen extraction site described	4	21
**Intra-operative Outcomes**		
Operating time	18	95
Estimated blood loss	15	79
Lymph node harvest	16	84
Total incision length	9	47
Distal resection margin or margin status	13	68
Positive margins	11	58
Intra-operative complications	0	0
**Post-operative Outcomes**		
Overall complication rate	19	100
Pain scores	5	26
Return to theatre	2	10
Length of hospital stay	16	84
Time to flatus/ bowel function	11	58
Recurrence rate	5	26
Follow-up duration	5	26

## Discussion

This scoping review demonstrates that although SP robotic colorectal surgery is technically feasible and appears safe in selected patients, outcome reporting remains fragmented and non-standardised. This work adds value to the current literature. Compared with the systematic review of Brucchi et al. (2025), which summarised perioperative and short-term safety outcomes, our work characterises the variability and gaps in outcome reporting across a range of clinically relevant domains [36]. Rather than synthesizing pooled estimates, we describe the degree to which studies differ in their methodological detail and completeness, thereby explaining why a meta-analysis remains premature for this field. In addition, we identified methodological and reporting gaps which can guide future research.

First, an imbalance in outcome domains was seen. The literature is heavily weighted toward technical and intra-operative metrics. Operative time, blood loss, and lymph node harvest are consistently reported, whereas patient-centered outcomes, including postoperative pain, functional recovery, cosmesis, and quality of life, are rarely evaluated. This imbalance reflects early-stage adoption focused on technical validation rather than comprehensive value assessment. SP surgery may offer unique theoretical advantages, such as improved cosmesis, potentially reduced pain, and patient preference for fewer incisions, but none of these factors were adequately assessed in the reviewed literature. Incorporating patient-reported outcome measures (PROMs) and experience measures (PREMs) into future research would help clarify whether patient choice and perceived recovery could justify wider adoption of SP techniques in visceral surgery.

In addition, long-term oncological outcomes remain insufficiently characterised. Follow-up is short, recurrence reporting inconsistent, and stage-specific analysis rare. Without durable oncological data, definitive conclusions regarding oncological equivalence to MP or laparoscopic surgery remain premature.

There is also little health system context. For example, reported length of stay appears longer than contemporary enhanced recovery benchmarks. At face value, these durations appear longer than those typically published for minimally invasive colorectal surgery [37]. However, system-level factors likely confound interpretation. Many included studies originated from South Korea whereby the nature of the public healthcare system does not prioritise early discharge or resource turnover. Conversely, in the United States, reimbursement models often provide a fixed payment for a 30-day episode of care regardless of hospital utilisation, creating strong incentives for rapid discharge. Variability in reimbursement models and discharge practices across countries and health systems complicates direct comparisons.

A further consideration is the influence of learning curve upon operative outcomes. There was a variability in operative metrics observed across studies, with the most notable being the wide range in operative time (119–357 min), and an outlier conversion rate of 29%. This may in part reflect differences in experience with the SP platform, rather than the technique itself. The SP is completely different from MP robotic surgery, with constrained instrument triangulation, a flexible camera system, and a compact operative field that requires platform-specific training. Existing evidence on the learning curve for SP robotic colorectal surgery remains limited. Available data from early-adopting centres suggest that operative efficiency improves substantially over an initial series of cases, with most authors reporting stabilisation of operative times after approximately 20–30 procedures [38], though formal learning curve analyses are limited. This is an important point when interpreting presentation of operative metrics. Future studies may report institutional case volume and surgeon experience with the SP platform to allow the reader to put results into context, and to enable learning curve-adjusted analyses.

Finally, there is a gap in economic evidence. No included studies assessed health economic outcomes or cost-effectiveness. The absence of such data reflects the early stage of SP robotic adoption. While initial procedural and capital costs are expected to be higher due to new technology and learning curves, potentially downstream benefits, such as shorter hospital stay or reduced complications, remain speculative As robotic technology continues to expand globally, value-based adoption requires integration of cost analysis, resource utilisation metrics, and long-term outcomes. Future prospective studies should integrate cost analysis to support value-based adoption.

We recognize the limitations in this work. The evidence is constrained by small, single-centre studies, often from a limited number of high-volume institutions in the United States and South Korea. While these institutions have pioneered SP robotic surgery, their outcomes may reflect unique expertise, patient selection and institutional protocols rather than broader applicability. Furthermore, overlapping datasets from these centres and retrospective designs introduce potential selection bias and reduce generalisability. Few studies reported follow-up beyond the short-term, and the absence of standardised definitions for complications and outcome metrics further hinders synthesis. Additionally, many studies were retrospective case series, at risk of selection bias and underpowered to detect clinically meaningful differences. Few adopted prospective or randomised designs, and even fewer reported long-term outcomes, limiting insight into the durability and safety of SP robotic procedures. Interpretation is further challenged by wide variation in methodology and reporting practices. Differences in patient selection (such as case complexity, BMI and co-morbidities) and in surgical technique (port placement, extraction site, angle of approach, and docking position) reduce reproducibility across centres. Outcome reporting is inconsistent, with non-standardised definitions and selective emphasis on statistically significant results, further hindering aggregation and interpretation. These challenges highlight the urgent need for a standardised approach to outcome measurement in SP robotic colorectal surgery.

Despite these limitations, the current literature provides valuable early insights. SP robotic colorectal surgery appears feasible and safe, with outcomes broadly comparable to MP approaches. The limitations reflect the early stage of adoption rather than flaws in the technique, and as more centres contribute data and reporting becomes standardised, the evidence base will mature.

The fragmented nature of reporting in SP robotic colorectal surgery underscores the need for a core outcome set (COS) tailored to this field. COS are consensus-driven sets of outcomes considered essential for reporting in all clinical studies. Developing a COS through a Delphi consensus involving surgeons, patients and other stakeholders would establish a minimum standardized dataset for reporting SP robotic colorectal surgery. Experience in other surgical specialities has shown that COS development, through consensus among surgeons, patients and other stakeholders, improves comparability and clinical relevance [39]. In SP CRS, this would enhance transparency, enable meaningful comparison, and facilitate future meta-analysis. Importantly, inclusion of PROMs, PREMs and health economic outcomes would align surgical research with patient priorities and healthcare sustainability.

## Conclusions

The current body of evidence on SP robotic colorectal surgery is characterised by non-standardised and incomplete outcome reporting. While early results suggest feasibility and safety, the inconsistency in reported variables prevents robust comparison with MP robotic techniques. Establishing a standardised reporting framework and a core outcome set – incorporating clinical, patient-reported, and economic measures – is essential to improve evidence quality, enable comparative research, and guide adoption of SP robotic systems in colorectal surgery.

## Data Availability

No datasets were generated or analysed during the current study.
